# Development of decision rules for an adaptive aftercare intervention based on individual symptom courses for agoraphobia patients

**DOI:** 10.1038/s41598-024-52803-z

**Published:** 2024-02-06

**Authors:** Maximilian Wilhelm, Markus Moessner, Silke Jost, Eberhard Okon, Volker Malinowski, Katharina Schinke, Sebastian Sommerfeld, Stephanie Bauer

**Affiliations:** 1 Center for Psychotherapy Research, Heidelberg University Hospital, Bergheimer Straße 54, 69115 Heidelberg, Germany; 2 Heidelberg University, Heidelberg, Germany; 3 German Center for Mental Health (DZPG), partner site Mannheim/Heidelberg/Ulm, Germany; 4 Median Zentrum für Verhaltensmedizin Bad Pyrmont, Median West GmbH, Berlin, Germany; 5 Median Parkklinik Bad Rothenfelde, Median Parkklinik Bad Rothenfelde GmbH, Berlin, Germany; 6 AHG Klinik Waren, Fachklinik Waren GmbH, Waren, Germany

**Keywords:** Psychology, Anxiety, Rehabilitation

## Abstract

As other mental illnesses, agoraphobia is associated with a significant risk for relapse after the end of treatment. Personalized and adaptive approaches appear promising to improve maintenance treatment and aftercare as they acknowledge patients’ varying individual needs with respect to intensity of care over time. Currently, there is a deficit of knowledge about the detailed symptom course after discharge from acute treatment, which is a prerequisite for the empirical development of rules to decide if and when aftercare should be intensified. Therefore, this study aimed firstly at the investigation of the naturalistic symptom course of agoraphobia after discharge from initial treatment and secondly at the development and evaluation of a data-driven algorithm for a digital adaptive aftercare intervention. A total of 56 agoraphobia patients were recruited in 3 hospitals. Following discharge, participants completed a weekly online monitoring assessment for three months. While symptom severity remained stable at the group level, individual courses were highly heterogeneous. Approximately two-thirds of the patients (70%) reported considerable symptoms at some time, indicating a need for medium or high-intense therapeutic support. Simulating the application of the algorithm to the data set resulted in an early (86% before week six) and relatively even allocation of patients to three groups (need for no, medium, and high-intense support respectively). Overall, findings confirm the need for adaptive aftercare strategies in agoraphobia. Digital, adaptive approaches may provide immediate support to patients who experience symptom deterioration and thus promise to contribute to an optimized allocation of therapeutic resources and overall improvement of care.

## Introduction

Anxiety disorders are highly prevalent and contribute substantially to the burden of disease^1,2^. Among anxiety disorders, agoraphobia is a common form with an estimated 12-month prevalence of 4.0^3^. Many individuals with agoraphobia also experience panic attacks in the feared or avoided situations^4^. Agoraphobia and panic disorder are associated with substantial impairment for the individual as well as high excess costs for the health care system^5–9^.

Cognitive behavioral therapy (CBT) has proven effective for the treatment of panic disorder and agoraphobia, and on average, patients benefit from it^10–12^. However, only about half of the patients achieve remission after treatment^13,14^. Further, a considerable proportion of patients experience residual symptoms at the end of CBT^15^. Long-term outcome is heterogeneous and relapses are common^7,16^. Pharmacological treatment or a combination with CBT is also an efficacious treatment option^16–19^, yet, its effects slowly diminishes over time when discontinued^17,20^. However, despite effective treatments, many patients face a challenging time after both psychological and pharmacological treatment. It is known for various mental disorders that the first months after treatment termination are a particularly difficult phase in which patients are confronted with the problems of daily life^21,22^. Maintenance treatment and aftercare interventions can help to reduce symptom severity, maintain treatment gains, and detect or prevent relapse^23,24^. However, existing aftercare interventions are only sufficient for a subgroup of patients^21,25,26^. Patients’ needs after treatment termination differ. In addition, symptom trajectories show large variation. As a consequence, interventions are needed that adjust the intensity of support to the patients’ current needs, i.e. their symptom severities.

In recent years, the idea of optimizing treatment by taking into account patients’ individual needs has received huge attention^27^. The basic idea of such *personalized treatment* approaches is to tailor [e.g. Ref.^28^] or choose [e.g. Ref.^29^] the optimal therapy for an individual among the available interventions in a *data-driven* way^30,31^. For example, personalization can refer to treatment *intensity* [e.g. Ref.^32^], treatment *packages* [e.g. Ref.^31^], or treatment *components* [e.g. Ref.^33^]. A common feature of these approaches is that decisions about the “right” intervention are made in the very beginning, based on predictions. In the area of depression and anxiety disorders, the exploration of central residual symptoms currently appears promising to improve prognostic models of relapse^34^. However, a reliable prediction of symptom courses is only moderately successful so far^35^, and thus it is not possible to administer specific aftercare interventions to individual patients e.g. depending on their (high versus low) risk of relapse. Therefore, the concept of adaptive treatment appears more suitable for the aftercare context, i.e. the implementation of an empirically derived algorithm that determines whether, when, and how treatment should be intensified over time^36–39^. Several components play a role in an adaptive approach: *Modifications* to the treatment (e.g. changes in treatment intensity) are guided by a-priori decision rules at specific *stages* (i.e. predefined time points) and consider *tailoring variables* (e.g. symptom level). For instance, a decision rule could suggest maintaining a low-intense treatment (no modification) if there is a decrease in avoidance behavior (tailoring variable) within the first three weeks of treatment (decision stage). Vice versa, in case of an increase in avoidance behavior, the frequency of psychotherapy sessions could be increased.

The decision rules in adaptive approaches are developed using empirical data (e.g. longitudinal self-report data) and appropriate statistical analyses^27^. By applying the a-priory defined decision rules, standardized and thus reproducible decisions can be made during the observation period^36^. One way to operationalize decision rules is to introduce *cutoff-scores,* i.e., a score that determines the level of symptom severity that must be exceeded to start with a more intense treatment alternative. Appropriate cutoffs must be set to keep the balance between a too low (i.e. treat all patients with the most intensive level) and a too high score (i.e. no patient receives the most intensive level of care). Furthermore, available resources and potential risks of modifications (e.g. side effects) should be considered when defining decision rules.

Whereas just a few years ago, data collection required great effort, new technologies such as smartphones, tablets, and personal computers make it relatively easy and cost effective to monitor the health condition of individual patients. By leveraging data, such as those collected through internet-based routine outcome monitoring^40,41^, treatment could be enhanced by delivering specific additional components at specific time points^36,42,43^. These components may also be provided via digital technologies. For several mental disorders, internet- and mobile-based interventions (IMIs) have shown promising results for maintaining treatment gains^22^. It is expected that IMIs will be more accessible and far less costly compared to traditional (face to face) aftercare interventions, and thus may find their way into routine care. Although combining the strengths of IMIs and adaptive approaches seems promising, these approaches must first be developed and empirically tested and then implemented to routine care.

In developing an adaptive intervention, a variety of critical decisions (e.g. definition of decision rules) must be made based on empirical (longitudinal) data^37^. However, despite its substantial impact on individuals and society, agoraphobia remains one of the least researched anxiety disorders^44^. Studies on post-treatment symptom trajectories and relapse are limited. Especially for routine care conditions there are hardly any data. Our knowledge on the natural course of symptoms after treatment termination is insufficient to establish meaningful decision rules for adaptive aftercare.

This study therefore pursued two aims. The first aim was to explore the natural symptom courses in patients with agoraphobia, with and without panic disorder, during the initial three months following treatment termination. The primary objective was to describe the natural symptom courses after discharge and assess the necessity for additional support. The second aim of the study was to develop algorithms (decision rules) for a potential adaptive aftercare intervention and to apply these decision rules to the collected data in order to test their plausibility and simulate the patients’ group allocations over time.

## Results

### Recruitment and adherence

Between Mai 2021 and December 2022, *N* = 60 inpatients were recruited. Three participants did not complete an assessment, and one had withdrawn consent to the study. Thus, four cases were excluded from the data analysis, and the final sample was *N* = 56. The number of completed assessments ranged from 4 to 13 (*M* = 12.04; *SD* = 2.22). Of the 56 participants, *N* = 49 provided data for baseline (t1) and *N* = 51 completed the final assessment (t2). Altogether, participants’ adherence was excellent, they completed 92.58% (674 of 728) of the scheduled assessments.

The mean age of participants was 45.36 years (*SD* = 10.35 years) ranging from 20 to 62 years. Most of the patients (64.00%) were female, no one identified as diverse. Average duration of inpatient treatment was 45.18 days (*SD* = 10.02; min = 31, max = 78). Out of the 56 patients 54 (96.00%) met ICD-10 diagnosis for agoraphobia with panic disorder, two patients met ICD-10 diagnosis for agoraphobia only. Regarding comorbidities, 39 patients (69.6%) were diagnosed with at least one additional disorder. Of these, 34 patients were diagnosed with an affective disorder (depressive episode (F32): n = 7; recurrent depressive disorder (F33): n = 27. At t1, n = 23 patients (46.9%) stated that they currently took medication for their anxiety disorder. Specifically, n = 14 stated that they were treated with selective serotonin reuptake inhibitors (SSRIs). Nine patients mentioned other antidepressants.

### Descriptive statistics

The observed means for all measures at baseline (t1) and end of study (t2) are shown in Table 1. While only 3.92% of patients indicated no clinically relevant symptoms on the Panic and Agoraphobia Scale [PAS; Ref.^45^] (PAS = 0–8) at t2, 37.25% reported mild (PAS = 9–18) and 37.25% reported moderate (PAS = 19–28) symptom severity. Moreover, 15.69% reported severe (PAS = 29–39) and 5.88% very severe (PAS ≥ 40) symptom severity. Further, 11.76% indicated normal (0–2), 50.98% mild (3–5), 25.49% moderate (6–8), and 11.76% severe (9–12) symptom severity on the total Patient Health Questionnaire-4 [PHQ-4; Ref.^46^] score at t2.

**Table 1:** Clinical outcome at baseline (t1), end of study (t2), and change (t2–t1).

Instrument (range)	t1 (n = 49)		t2 (n = 51)		Change (t2–t1; n = 46)	
	M	SD	M	SD	M	SD
Panic and agoraphobia scale (0–52)	19.41	9.15	21.24	9.89	0.43	7.44
Patient health questionnaire (0–12)	4.96	3.04	5.06	2.83	− 0.07	2.26
Overall anxiety severity and impairment (0–20)	10.70	4.30	9.67	3.97	− 1.38	3.20
Mobility inventory (1–5)—alone	2.79	0.81	2.85	0.86	− 0.02	0.52
Mobility inventory (1–5)—accompanied	2.25	0.65	2.22	0.71	− 0.06	0.50
Agoraphobic cognitions (1–5)	2.21	0.64	2.26	0.54	0.00	0.46
Body sensations (1–5)	2.94	0.78	2.91	0.67	− 0.07	0.53
Generalized anxiety (0–21)	9.78	5.10	10.41	4.36	0.37	2.89

The observed means for the weekly monitoring are shown in Table 2. On a group level, the symptom burden for the Overall Anxiety Severity and Impairment Scale [OASIS; Ref.^47^] was quite stable, it ranged between *M* = 8.94 in week 6 (*SD* = 4.07) and week 10 (*SD* = 4.52) and *M* = 10.70 (*SD* = 4.30) at baseline. For the avoidance item of the OASIS (item 3), patients reported least avoidance in week 10 (*M* = 1.62; SD = 1.01) and highest avoidance directly after discharge (*M* = 2.08; *SD* = 1.03). Only two patients reported not having a single panic attack during the observation period. On average, patients experienced between *M* = 1.29 (*SD* = 2.10; week 12) and *M* = 0.91 (*SD* = 1.08; week 2) panic attacks per week. The total score on the PHQ-4 ranged between *M* = 4.42 (*SD* = 2.88; week 10) and *M* = 5.67 (*SD* = 2.89; week 3).

**Table 2:** Observed means for the weekly monitoring assessment.

Instrument	Week													
		0	1	2	3	4	5	6	7	8	9	10	11	12
	*n*	49	52	55	55	53	52	52	49	52	52	52	51	51
OASIS (0–20)	*M*	10.70	9.13	9.07	9.80	9.62	9.33	8.94	9.49	9.08	9.29	8.94	9.53	9.67
	*SD*	4.30	4.25	3.50	3.68	3.62	3.87	4.07	4.34	4.04	4.06	4.52	4.06	3.97
Avoidance (0–4)	*M*	2.08	1.75	1.64	1.75	1.85	1.69	1.65	1.73	1.71	1.75	1.62	1.80	1.88
	*SD*	1.03	1.03	0.91	0.91	0.93	0.81	0.93	1.00	0.89	0.93	1.01	0.92	0.97
PA (0–11)	*M*	1.26	1.00	0.91	1.24	1.06	0.98	1.06	1.20	0.98	0.96	0.96	1.20	1.29
	*SD*	1.79	1.62	1.08	1.36	1.86	1.38	1.82	1.96	1.26	1.80	1.48	1.90	2.10
PHQ-4 (0–12)	*M*	4.96	4.73	4.65	5.67	5.19	4.71	4.92	5.27	4.65	4.75	4.42	4.8	5.06
	*SD*	3.04	2.79	2.79	2.89	3.04	2.89	2.92	3.03	2.56	2.79	2.88	2.74	2.83

*PA* panic attacks.

At t2, patients were asked about perceived difficulties related to the transition from inpatient treatment to their everyday lives and also about the utilization of professional support during the observation period. In response to the question “Have you sought professional help for your agoraphobia since your discharge from the hospital?”, 25 of the 51 patients (49%) stated that they had sought some sort of professional help after discharge. Specifically, 22 patients initiated outpatient psychotherapy. The average gap between the start date of help (actual and planned) was approximately 40 days (*M* = 39.5; *SD* = 29.9; min = 1; max = 106; n = 28). When asked "Did you find the transition from hospital to everyday life difficult?" 5.9% answered "not at all", 17.6% "not so much", 41.2% “rather”, and 35.3% "very much".

### Algorithm development

For the development of the adaptive aftercare intervention, we assumed that the main goal is to maintain treatment gains, to support with symptom exacerbations, and react to relapses and crises in order to allocate the resources where they are needed most. We defined 3 levels of treatment intensities (level I: minimal intervention, e.g. psychoeducation, level II: low-intensity intervention, e.g. online exercises, level III: high-intensity intervention, e.g. crisis management). Based on the symptom severity measured in the weekly monitoring (tailoring variables), each week a decision is made (via a priori decision rules) to increase the level of support if needed. Moreover, the decision rules should allow for meaningful adjustments over time. In doing so, the allocation into the three intervention levels should be made timely. A cutoff-based strategy was chosen to capture the clinical approach of an initial low intensity treatment (watchful waiting) and to allow for adjustments in intensity over time if a cutoff is exceeded^36^. This strategy is intended to prevent more intensive and costly treatment from being carried out unnecessarily and allocate resources to those patients who need it most.

**Figure 1:**
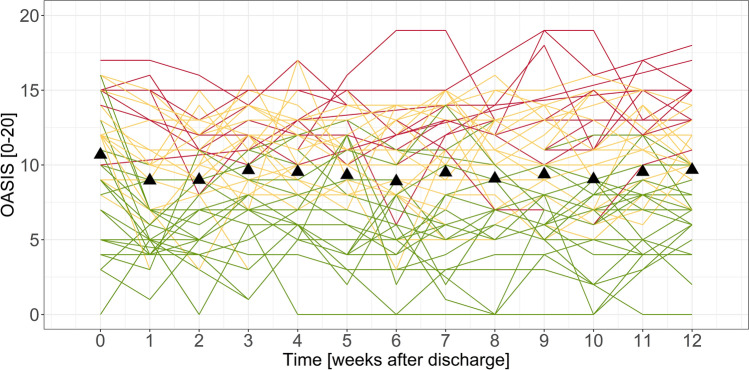
Individual symptom trajectories measured with the sum score of the OASIS. Triangles represent the mean values. The three groups indicated by colors correspond to the PAS level at the end of the study: red = severe, orange = moderate, green = mild.

The severity of panic and agoraphobia, measured by the total score of the PAS at the final assessment, was used as a criterion for post-inpatient health status. Based on the severity of agoraphobia symptoms 3 months after discharge, patients were divided into three subgroups: Patients indicating no clinically relevant or mild symptoms (41.17%), moderate symptoms (37.25%), and severe symptoms (21.57%). Differences in symptom courses between the 3 classes were analyzed. To account for the individual courses over time, the weekly monitoring instruments were analyzed using descriptive statistics and data visualization techniques. Figure 1 displays individual symptom trajectories for the three subgroups. This procedure was repeated for the avoidance item, the panic attacks item, and the total score of the PHQ-4.

Regarding the definition of the cutoff-scores, the descriptive and visual analysis of the individual courses revealed distinct patterns within the 3 subgroups. Subgroup 1 reported low symptoms (examples based on mean OASIS scores over all patients over all measurement points: *M* = 5.88) with some variation (*SD* = 2.93) over the complete observation period. Subgroup 2 experienced mild symptoms (*M* = 9.78, *SD* = 2.89) throughout the twelve weeks. Subgroup 3 showed on average higher symptoms with additional symptom peaks (*M* = 12.31, *SD* = 3.31). However, the most straightforward way, the introduction of two cross-sectional cutoffs (one for each level), did not work out, as over the course of the twelve weeks, almost every patient reported at least one difficult week. Therefore, two different cutoff scores for the algorithm were defined: *Cutoff 1*, based on symptom severity over two consecutive weeks, and *Cutoff 2*, based on symptom severity at a single point in time. A moderate continuous symptom severity (Cutoff 1) may indicate the need for additional support, while a single extreme value (Cutoff 2) may suggest an acute crisis that requires immediate attention and support. Following an iterative process comparing mutual interdependencies of the decision rules and taking into account clinical considerations, the final cutoff-scores shown in Table 3.

**Table 3:** Proposed cutoff-scores and proportion of exceeded cutoffs.

	OASIS		Avoidance		Panic attacks		PHQ-4	
	Score	Exceeded (n)	Score	Exceeded (n)	Score	Exceeded (n)	Score	Exceeded (n)
None		39.3% (22)		53.6% (30)		71.4% (40)		50.0% (28)
Cutoff 1	11.0	50.0% (28)	2.5	33.9% (19)	2.0	14.3% (8)	6.0	14.3% (8)
Cutoff 2	16.0	10.7% (6)	3.0	12.5% (7)	4.0	14.3% (8)	8.0	35.7% (20)

Cutoff 1 refers to symptom severity over two consecutive weeks (mean).

### Algorithm simulation

**Figure 2:**
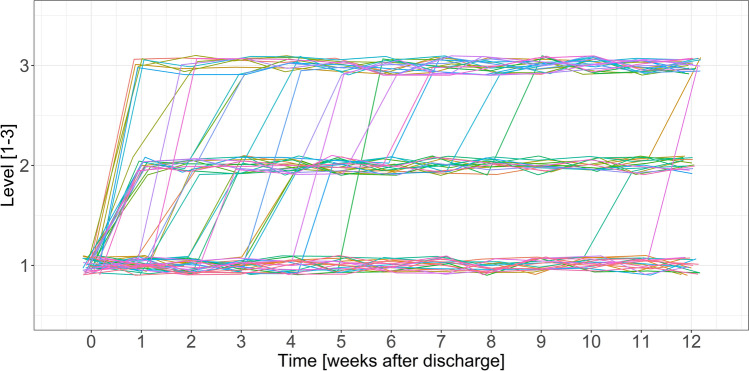
Adaptive group allocation over the course of 12 weeks after hospital discharge.

To explore the performance of the developed decision rules, we simulated the patients’ group changes over time based on their data. The application of the decision rules and the transitions into more intense treatment groups are shown in Fig. 2. All patients start with minimal intervention and can then be upgraded in a more intensive treatment level as they progress. Within the simulation, the allocation of patients occurred relatively evenly across the three proposed levels. At t2, 30.4% (n = 17) of patients did not exceed either cutoff, 26.8% (n = 15) exceeded Cutoff 1, 42.9% (n = 20) reported severe symptoms for at least one week, exceeding Cutoff 2. Further, 44 of 51 group allocations (86.3%) took place before week 6, i.e., all patients could benefit from the higher intensity for a significant period of time. Finally, the algorithm allowed for sequential adaptions as patients moved from level I to level II or from level II to level III or from level I directly to level III. In addition to the cutoff-scores, Table 3 shows the proportion of exceeded cut-offs. Cutoff 2 on the PHQ-4 (8.0) was exceeded more often (20 times) than the other constructs (OASIS: 6 times, avoidance: 7 times, panic attacks: 8 times). Raising the PHQ-4 Cutoff 2 value from 8 to 9 would result in 15 (26.8%) instead of 20 (35.7%) patients exceeding Cutoff 2 and overall, 20 (35.7%) instead of 24 (42.9%; one case exceeded another Cutoff 2 score besides the PHQ-4 Cutoff 2). For the final decision rules, however, we maintained the PHQ-4 categories defined by the authors [normal 0–2, mild 2–5, moderate 6–8, severe 9–12; Ref.^46^].

## Discussion

This longitudinal multicenter study explores naturalistic symptom progression in patients with agoraphobia with and without panic disorder, for three months after hospital discharge. Two thirds of patients still show considerable symptoms at the end of inpatient treatment. Likewise, 76.5% rated the transition period after discharge rather or very difficult, clearly highlighting the need for additional support after discharge from inpatient treatment. Although around half initiated outpatient treatment, they face a gap of around 40 days without help. The results confirm that consecutive aftercare offers are meaningful. Adaptive interventions seem especially promising as they can take into account the heterogeneous needs and symptom courses in this critical transition period. The resources saved by the internet-based and adaptive components could lead to higher acceptance and lower cost per person, increasing the likelihood of implementation in routine care.

The feasibility of the weekly symptom monitoring proved to be excellent; while only 3 patients did not participate, the remaining patients completed approximately 93% of the assessments. The high adherence attests the patients’ interest and the need for additional support directly after discharge. This opens the perspective for further approaches based on longitudinal data collection, for example longer observation periods or more intense assessment schedules [e.g. ecological momentary assessment; Ref.^48^].

Finally, based on the monitoring data, decision rules for a possible adaptive aftercare intervention are derived. While the group-level analyses are not very informative (no significant variation at the group level), individual symptom courses show very diverse patterns over time. Descriptive and visual analyses of individual symptom courses confirm high heterogeneity of symptom courses after discharge. Symptom courses are very heterogeneous within individuals, suggesting that some patients go through a difficult transition period after discharge from hospital, while others manage to stabilize their treatment gains. Using a cutoff-based strategy, an adaptive aftercare intervention is sketched out. Final decision rules incorporate two different cutoff scores on central monitoring measures, that performed well in identifying patients in need quickly. Cutoff 1 is based on mean symptom severity over two consecutive weeks, and Cutoff 2, is based on a single measurement point.

The simulation of the decision rules results in a reasonable group allocation over time. First, with about 70% of patients upgraded to a more intensive level of support, valuable (therapeutic) resources would also be saved. In an internet-based environment, minimum support could be highly automated [e.g. supportive monitoring; Ref.^21^] and more intense support could be provided in therapeutic contact [e.g. expert-chat; Ref.^49^]. In addition, especially patients indicating high symptom severity (after all, 43% of patients) could benefit from the resources that are freed up. Second, with about 86% of adjustments take place in the first weeks after discharge from hospital, patients could profit from the higher intensity of support significantly. The weekly symptom monitoring, combined with adaptive internet-based interventions, enables for rapid response to deterioration and immediate provision of additional help.

This study has several strengths. First, the collection of data within patients’ daily lives provides naturalistic and valuable insights into individual symptom courses following inpatient treatment. Recruiting at different clinics increases the study’s validity. Second, with the large number of completed assessments over 13 measurement points, data quality is very high. It demonstrates that routine outcome monitoring is a feasible tool to inform (adaptive) aftercare. This is consistent with findings that patients support routine outcome monitoring when the purpose is clear, and implementation is done with care^40^. Third, the adaptive allocation algorithm proves plausible and promises to facilitate resource allocation. Over the tree months, the majority of patients (70%) experience a crisis. Predicting who will experience a crisis and when is challenging. Therefore, continuous symptom monitoring is crucial to individualize adaptive aftercare interventions.

The sample size for the development of an algorithm is rather small, potentially limiting the generalizability of the derived decision rules. While the transition period in an inpatient context could be particularly challenging, relapses or residual symptoms of anxiety disorders at the end of CBT treatment are independent of country and setting. Given that the study's findings predominantly pertain to the inpatient context, future research should also explore outpatient settings. The study's monitoring and adaptive aftercare procedure is applicable across various contexts (bridge waiting periods and support individuals during/after inpatient or outpatient therapy). Moreover, the efficacy and superiority of adaptive interventions compared to conventional aftercare approaches remains to be investigated^22^. It is also not possible to examine within this study what effect an aftercare intervention would have had on the course. Due to a possible improvement in the course of symptoms, fewer patients would be expected in the most intensive treatment group. However, the aim of the study is to outline such an algorithm for routine care that can and must be validated in a larger randomized study^36^. Also, no information on the effectiveness of the initial inpatient treatment was available.

The novel advances in technology are promising, as they can overcome some of the challenges of the face to face model of psychotherapy^50^ especially for patients with mild symptoms. Internet-based care services are independent of time and setting and can be offered at relatively low cost. The potential of modern technology is far from exhausted. Especially at the transitions between services and/or sectors, e.g. waiting times for treatment^51^, and aftercare or relapse prevention^21,22,26^.

## Conclusion

Patients with agoraphobia require support after end of acute treatment. However, patients experience heterogeneous symptom courses and consequently require individualized treatment strategies. Personalized, adaptive treatment strategies can adjust the level of support dynamically over time to better fit the individual needs to the intensity of support. Especially during crisis. Internet-based adaptive aftercare approaches are feasible and have great potential to improve the healthcare situation by allocating resources to the patients who need it most when they need it most.

## Method

### Participants and setting

Participants were recruited from three psychosomatic clinics (MEDIAN Center for Behavioral Medicine Bad Pyrmont, AHG Psychosomatic Hospital Waren, MEDIAN Clinic Bad Rothenfelde) where they received inpatient psychotherapy treatment. Inpatient treatment integrated guideline-based^18^ psychotherapy and pharmacotherapy. Psychotherapy, grounded on cognitive behavioral therapy (CBT) principles, incorporates psychoeducation, cognitive restructuring, coping skills, and exposure exercises (guided and self-managed). CBT is offered in both individual and group settings. While undergoing inpatient treatment, patients were encouraged by therapists to consider outpatient care upon their discharge from the hospital. Given the division between inpatient and outpatient services in Germany, it becomes the responsibility of the patient to actively seek additional care.

Inclusion criteria were age above 18 years, inpatient therapy in one of the cooperating clinics, internet access, a valid e-mail address, as well as a clinical diagnosis of agoraphobia with or without panic disorder. The clinical diagnosis was made within the standard diagnostic procedures of the respective clinics according to the 10th version of the classification of mental and behavioural disorders [ICD-10; Ref.^52^] guidelines. The diagnostic criteria of the ICD-10 are as follows: (a) a marked and persistent fear of, or avoidance of, several situations; (b) exposure to the phobic situation almost invariably provokes an immediate anxiety response; (c) significant emotional distress due to avoidance or symptoms; (d) symptoms are restricted to the feared situations or thoughts about them; (e) the anxiety or phobic avoidance is not better accounted for by another mental disorder. A panic disorder can be indicated with the fifth digit (F40.00 Agoraphobia without panic disorder; F40.01 Agoraphobia with panic disorder). Exclusion criteria included acute suicidality, insufficient German language proficiency, and substance abuse. Under routine conditions, there were no restrictions on the use of other psychological or pharmacological treatment for the duration of the study. Patients provided written informed consent. The study was performed in accordance with the declaration of Helsinki, it was approved by the ethics committee of the University Hospital Heidelberg, Germany.

### Procedure

Participants were asked to complete a weekly online monitoring survey for three months following discharge from inpatient psychotherapeutic treatment. Once a week, participants received an e-mail with a hyperlink to the online survey. Assessments were conducted using the Software *Assessment and monitoring of mental health* [ASMO; Ref.^41^]. If the assessment was not completed after 3 days, participants received an automated e-mail reminder. If participants failed to complete the assessment twice in a row, they were contacted by phone to resolve technical issues. Participants who completed the final assessment received an online gift voucher (€30).

### Assessment of self-reported symptom severity

Between baseline assessment at discharge (t1) and the end of the observation period (t2), core symptoms of agoraphobia and depressive and generalized anxiety symptoms were assessed at weekly monitoring assessments (WM). Questions concerning basic demographic variables (age, sex) and medication were included at t1. Questions on the use of psychological treatment and the transition period between hospital dismission and everyday life were asked at t2. All assessments were conducted as online self-report. Table 4 shows the instruments used at different points in time.

**Table 4:** Overview of instruments used at different points in time.

Variable	Instrument	t1	WM	t2
Demographics		X		
Panic and agoraphobia severity	Panic and agoraphobia scale (PAS)	X		X
Severity indicator for generalized anxiety disorder	Generalized anxiety disorder scale (GAD-7)	X		X
Avoidance behaviour	Mobility inventory (MI)	X		X
Agoraphobic cognitions	Agoraphobic cognitions questionnaire (ACQ)	X		X
Body sensations	Body sensations questionnaire (BSQ)	X		X
Severity of anxiety symptoms and treatment adherence	Jena anxiety monitoring list (JAMol)	X	X	X
Depression and anxiety	Patient health questionnaire (PHQ-4)	X	X	X

*t1* baseline, *WM* weekly monitoring, *t2* end of study after 3 months.

#### Panic and agoraphobia scale (PAS)

The German version of the PAS^45^ was conducted to measure the severity of the disorder. The PAS comprises 13 items with five response options each (0 to 4). The total score consists of the sum of all items (range 0 to 52). The internal consistency of the PAS is good, Cronbach's α = 0.86^40^.

#### Generalized anxiety disorder scale 7 (GAD-7)

The GAD-7^53^ measures generalized anxiety disorder and the severity of generalized anxiety using 7 items that range on a four-point Likert scale from *not at all* (0) to *almost every day* (3). The internal consistency of the GAD-7 is very good, Cronbach’s α = 0.85^54^.

#### Body-related anxiety, cognitions, and avoidance questionnaire (AKV)

The AKV^55^ is the translated version of the *Agoraphobic Cognition Questionnaire* (ACQ) and the *Body Sensations Questionnaire* [BSQ; Ref.^56^], and the *Mobility Inventory*^57^. The combination of the three questionnaires is widely used and allows the examination of central anxiety constructs. The *ACQ* comprises 14 items on a 5-point Likert scale ranging from *never* (1) to *always* (5) to measure the frequency with which anxiety-related cognitions occur when the person is nervous or anxious. The internal consistency of the ACQ is acceptable, Cronbach's α = 0.79. The *BSQ* consists of 17 items and measures the extent of anxiety about physical symptoms (e.g. *weak knees*) on a 5-point Likert scale ranging from *not at all* (1) to *extremely* (5). The total score is calculated from the mean of all items answered. The internal consistency of the BSQ is good, Cronbach's α = 0.87. The *MI* uses 27 items to assess the extent of avoidance behavior in various situations (e.g. *riding buses*). The data are collected both alone and in company on a five-point Likert scale from *never* 1 to *always* 5. In the evaluation, the total score is composed of the mean scores of the *avoidance alone* and *avoidance accompanied* scales. The internal consistency of both scales of the MI is excellent, Cronbach's α = 0.96.

#### Jena anxiety monitoring list (JAMoL)

The first two modules of the JAMoL^58^ were used to assess the severity of anxiety symptoms (items 1–6) and the patient’s adherence to therapy (items 7, 9, 10) on a weekly basis. The first five items of the anxiety symptoms module [using the German version of the Overall Anxiety Severity and Impairment Scale; OASIS-D; Ref.^47^] measure the frequency and intensity of anxiety (e.g. *How often were you anxious in the past week?*), avoidance behaviors, and resulting impairments for the past week. Item 6 captures the frequency of panic attacks (response options 0–10 covered the exact number of panic attacks experienced, 11 served as > 10). While item 7 measures the frequency of exposure exercises, item 9–10 addresses the severity of perceived anxiety during the exercise. The internal consistency of the English-language version of the OASIS is good, Cronbach's α = 0.80^59^.

#### Patient health questionnaire-4 (PHQ-4)

The PHQ-4^46^ consists of four items and measures core depression and anxiety symptoms on a four-point Likert scale, ranging from *not at all* (0) to *almost every day* (3). The total score is an overall measure of symptom burden, the internal consistency is good with Cronbach's α = 0.85.

### Sample size and statistical analyses

We aimed to recruit *N* = 60 patients. This sample size allows the frequency of agoraphobic avoidance behavior and other outcome measures to be assessed with sufficient accuracy, which is appropriate for the study objective.

To describe the naturalistic symptom course of patients with agoraphobia with and without panic disorder over three months after treatment termination, descriptive statistics of outcomes on group and individual level were reported. Change in symptoms were calculated (t2–t1). Based on weekly symptom courses, decision rules were deducted and tested for an adaptive aftercare intervention. Decision rules were constructed to match the aftercare intervention’s intensity to the participant’s symptom severity three months after hospital discharge, based on the total PAS score. All statistical analyses were performed in R [Version 4.1.2; Ref.^60^].

## Data Availability

The data that support the findings of this study are available from the corresponding author upon reasonable request.
